# Transcriptome and Metabolome Analysis of the Mechanism of Environmental Adaptability in *Populus* Roots

**DOI:** 10.3390/plants14172691

**Published:** 2025-08-28

**Authors:** Panrui Chen, Jiaxin Luo, Qiushuang Zhao, Miao Yu, Xiaona Pei, Luping Jiang, Rui Han, Xiyang Zhao

**Affiliations:** 1 Jilin Provincial Key Laboratory of Tree and Grass Genetics and Breeding, College of Forestry and Grassland Science, Jilin Agricultural University, 2888 Xincheng Street, Changchun 130118, China; 15396062892@163.com (P.C.); 15834905679@163.com (J.L.); 18346376063@163.com (M.Y.); 2 State Key Laboratory of Tree Genetics and Breeding, Northeast Forestry University, 26 Hexing Road, Harbin 150040, China; zhaoqs1002@163.com; 3 College of Horticulture, Jilin Agricultural University, 2888 Xincheng Street, Changchun 130118, China; xiaonapei2020@163.com

**Keywords:** (*P. simonii* × *P. nigra*) × *P. deltoides*, transcriptome, metabolome, environmental adaptability

## Abstract

Poplar (*Populus* spp.) is a keystone commercial tree species in Northeast China, valued for its high economic returns. The genotype-by-environment (G × E) interaction critically governs its growth performance and ecological adaptability, which are pivotal for ensuring the long-term sustainability and economic viability of poplar plantations. In this study, the fibrous roots of the (*P. simonii* × *P. nigra*) × *P. deltoides* clone planted at three distinct sites, including Lishu (named SR1), Xinmin (named SR2), and Cuohai (named SR3), were used to perform transcriptome and metabolome. Comparative analysis revealed 6246, 3455, and 3854 differentially expressed genes (DEGs) in the SR1 vs. SR2, SR1 vs. SR3, and SR2 vs. SR3 comparisons, respectively. These DEGs were functionally enriched in pathways associated with antioxidant enzyme activity, stimulus response, plant hormone signal transduction pathways, and α-linolenic acid metabolism. Metabolomic analysis identified 106, 147, and 189 significantly differentially accumulated metabolites (DAMs) across the same comparisons, primarily linked to glutathione metabolism, butanoate metabolism, and pentose–glucuronate interconversions. Notably, we identified a core regulatory module comprising 57 genes and four key metabolites within the α-linolenic acid metabolic pathway, which exhibited strong correlations with phenotypic adaptability. These findings provide mechanistic insights into poplar’s plasticity under environmental heterogeneity, offering a molecular roadmap for future breeding strategies and the sustainable expansion of poplar cultivation.

## 1. Introduction

The growth of tree species is governed by both genetic and environmental factors. Notably, even trees with identical genotypes may exhibit phenotypic divergence across different regions due to significant geographical heterogeneity [1,2]. In the context of breeding technology development, genetic–environment (G × E) interactions remain a focal point, as studies have demonstrated that environmental effects exert stronger influences on quantitative traits (e.g., growth and wood properties) than on qualitative traits (e.g., environmental adaptability) [3]. For comprehensive analysis, the quantitative traits, such as growth traits, wood properties, and quality traits, such as environmental adaptability, are often evaluated together [4,5]. Unlike annual crops, trees face prolonged breeding cycles, rendering them more susceptible to external environmental fluctuations. Poplar is primarily a native tree species in Northeast China, which can affect the regional ecological and economic benefits. Consequently, the selection of poplar with rapid growth and strong adaptability is significant for breeding [6]. Many regional experiments are conducted to explore the interaction between genetic and environmental effects of trees, thereby obtaining the adaptability of trees to the sites or conditions, including poplar [7]. For example, three hybrid poplar clones were planted under drought treatments (none, medium, and severe) over two years, and their growth and water use exhibited diversities [8]. A Nordic-Baltic study of 63 *Populus trichocarpa* clones across three sites revealed pronounced clonal plasticity in response to environmental gradients [9]. Similarly, studies on poplar, eucalyptus (*Eucalyptus* spp.), and black locust (*Robinia pseudoacacia*) hybrids further corroborate that field performance and productivity are heavily contingent on cultivation environments [10,11].

Regarding climatic factors, global studies establish a strong correlation between poplar mean annual increment (MAI) and precipitation, with increased rainfall enhancing biomass accumulation in U.S. and European plantations [12]. In arid regions, optimized irrigation and planting density boost productivity. Genomic approaches, such as QTL mapping, have identified drought-tolerance genes, facilitating the development of resilient transgenic cultivars [13]. Beyond precipitation, water use efficiency is also modulated by relative humidity and wind speed. For instance, low humidity accelerates transpiration, inducing water stress, while high winds promote leaf evaporation and trigger stomatal closure, reducing CO_2_ uptake and photosynthesis. Similarly, temperature plays a critical role in regulating growth phenology and physiology. In *Populus tremula*, warming advances bud break and delays dormancy, extending the growing season [14]. Moreover, hybrid poplar (*P. maximowiczii × P. nigra*) exhibits photosynthetic acclimation, shifting its optimum temperature (Topt) from 23 °C to 33 °C [15]. Furthermore, soil nutrients serve dual roles as essential elements for plant growth and as regulators of growth performance through their influence on soil microbial communities, plant physiological metabolism, and nutrient uptake efficiency [16]. Field studies demonstrate that integrated drip irrigation and fertilization systems significantly enhance poplar growth and wood volume accumulation. Notably, a balanced nitrogen–phosphorus–potassium (NPK) supply enhances both defensive metabolite accumulation and stress resistance in poplar [17]. Collectively, these findings underscore the importance of G × E interactions in enhancing our understanding of tree environmental adaptability. Tree species or clones selected with good performance are beneficial for commercial deployment and future breeding, also being the crucial parts of afforestation and introduction [18].

The root system constitutes approximately 20% of a tree’s biomass and holds significant potential as a bioenergy resource [19]. Functionally, roots are the primary organs of plants for coping with low nutrient availability and environmental stresses, adapting to their surroundings by perceiving external environmental signals and eliciting plastic responses in both morphology and physiology [20]. For instance, under mild N deficiency, trees adopt a “systemic foraging” strategy with a characteristic increase in root size to pursue nutrients from a distance [21]. Given these adaptive roles, elucidating root stress responses and their underlying physiological mechanisms is critical for advancing plant breeding strategies [22]. However, the subterranean nature of roots poses challenges for direct observation, resulting in limited research output and the absence of a unified theoretical framework for root-environment interactions [23,24]. While traditional studies relied on growth traits (e.g., Plant Height and Leaf Area) and physiological parameters (e.g., Photosynthetic rate and Chlorophyll content) [25,26], the advent of multi-omics technologies now enables systematic dissection of molecular responses.

Transcriptomics identifies key genes governing growth, metabolism, and stress responses [27]. For example, *PdNF-YB21* overexpression in poplar roots upregulated S-lignin biosynthetic genes, enhancing lignification and root growth [28]. Similarly, drought-responsive genes in *Populus davidiana* were enriched in hormone signaling, anthocyanin biosynthesis, and sugar metabolism pathways [29], while defense-related genes in *P. cathayana × canadensis* ‘Xinlin 1’ targeted MAPK signaling and flavonoid biosynthesis to counter *Hyphantria cunea* infestation [30]. Metabolomics reveals metabolic reprogramming during stress acclimation [31]. Phenolic compounds, flavonoids, and unsaturated fatty acids serve as biomarkers for induced resistance to *H. cunea* in poplar [32], while volatile organic compounds (e.g., 3-hexenal, trans-α-farnesene, 5-methyl-2-furanone) distinguish aphid- and moth-infested *P. tremula* leaves [33]. Transcriptomic and metabolomic analyses are the effective means to explain the internal changes caused by the environment. This comprehensive analysis method has been used to report the adaptation of *P*. × *xiaohei* T. S. Hwang et Liang under exogenous nitrogen treatment, three poplar varieties (*P. deltoides*. ‘Zhonglin 2025’, *P.* × *Euramericana*. ‘74/76’, and *P*. tomentosa cv ‘henan’) under bacterium *Lonsdalea populi* treatment, *P. balsamifera* under drought, and others [34,35,36].

In this study, we investigated the genotype–environment interactions in *(P. simonii × P. nigra) × P. deltoides* using roots from three ecologically distinct sites (SR1–SR3) with divergent temperature, precipitation, and soil elemental composition. The results of transcriptomic and metabolomic analyses were analyzed to explore the gene and metabolite profiles against the remarkable environmental difference. The results would select the appropriate cultivation area for the *(P. simonii × P. nigra) × P. deltoides* clone, which also provide a foundation for the research on the mechanism of genotype–environment interaction. The DEGs (differential expression genes) and DAMs (differentially accumulated metabolites) identified are important references for the understanding of promoting the poplar adaptability.

## 2. Results

### 2.1. Environmental Differences Among the Three Sites

**Figure 1:**
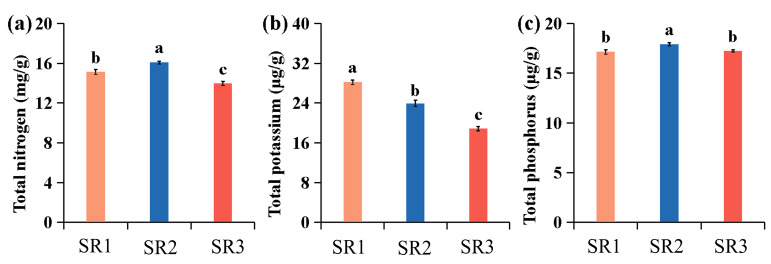
The soil element content in SR1, SR2, and SR3. (**a**) The total nitrogen content; (**b**) the total potassium content; (**c**) the total phosphorus content. To assess statistically significant differences, Duncan’s Multiple Range Test (IBM SPSS Statistics v26.0 software) was performed. Error bars represent the standard deviation of the means at *n* = 3. Significant differences (*p* < 0.05) are shown by distinct lowercase letters.

The soil element content among three sites (SR1, SR2, and SR3) was detected, and the results showed that SR2 had significantly higher levels of total nitrogen and total phosphorus compared to SR1 and SR3 (*p* < 0.05) (Figure 1a,c), while SR1 had the highest level of total potassium (Figure 1b). Synthetically, SR2 exhibited the highest soil nutrient content, and SR3 exhibited the poorest.

Analysis of meteorological data revealed significant climatic divergence among the three study sites. Specifically, SR2 exhibited the highest mean annual precipitation (600 mm), followed by SR1 (480 mm) and SR3 (465 mm). Mean annual temperature showed significant variation, with SR2 maintaining the warmest annual mean (8.2 °C), compared to SR1 (6.9 °C) and the substantially cooler SR3 (3.4 °C). Wind speed measurements demonstrated relative consistency across sites, ranging from 3.52 m/s (SR3) to 3.83 m/s (SR1). Relative humidity displayed a decreasing gradient from SR1 (61.88%) to SR3 (56.54%). All climate data were obtained from the China Meteorological Administration database URL: https://m.data.cma.cn/site/subjectDetail/id (accessed on 1 February 2023). Furthermore, morphological analysis demonstrated that the (*P. simonii* × *P. nigra*) × *P. deltoides* clone grown at the SR2 site exhibited significantly greater tree height and diameter at breast height (DBH) compared to the other two sites (Table 1).

### 2.2. The Quality Evaluation of Transcriptome and qRT-PCR Verification

**Figure 2:**
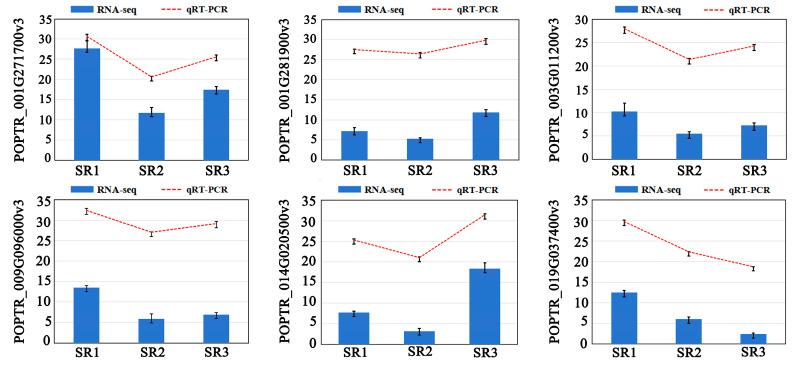
The expression of genes detected by qRT-PCR and RNA-seq. The y-axis on the left indicates relative expression levels of genes detected by qRT-PCR and the FPKM values of genes from RNA-seq data. To assess statistically significant differences, Duncan’s Multiple Range Test (IBM SPSS Statistics v26.0 software) was performed. Error bars represent the standard deviation of the means at *n* = 3.

We first conducted a comprehensive evaluation of transcriptome data quality. A total of 62.09 Gb Clean Data was obtained from nine samples, and each sample was more than 6.5 Gb. The GC content varied from 43.14% to 44.11%, and all Q30 values exceeded 90.52%. The alignment of Clean Reads against the *Populus simonii* reference genome was from 77.49% to 85.65% (Table S1). The number of 3283 new genes was found from the transcriptome (Table S2). The expression patterns of 6 randomly selected DEGs were evaluated in SR1, SR2, and SR3 by qRT-PCR using gene-specific primers to validate the accuracy and reliability of RNA-seq data (Figure 2). The comparative results demonstrated that the expression patterns of the 6 genes identified by qRT-PCR were consistent with those from RNA-seq sequencing, further showing the reliability of RNA-seq data. (Clean Data: Refers to the high-quality dataset derived from raw sequencing data through primary quality control. Clean Reads: Represent the de-replicated and deduplicated unique alignable sequences processed from Clean Data).

### 2.3. The Analysis of Differential Expression Genes

**Figure 3:**
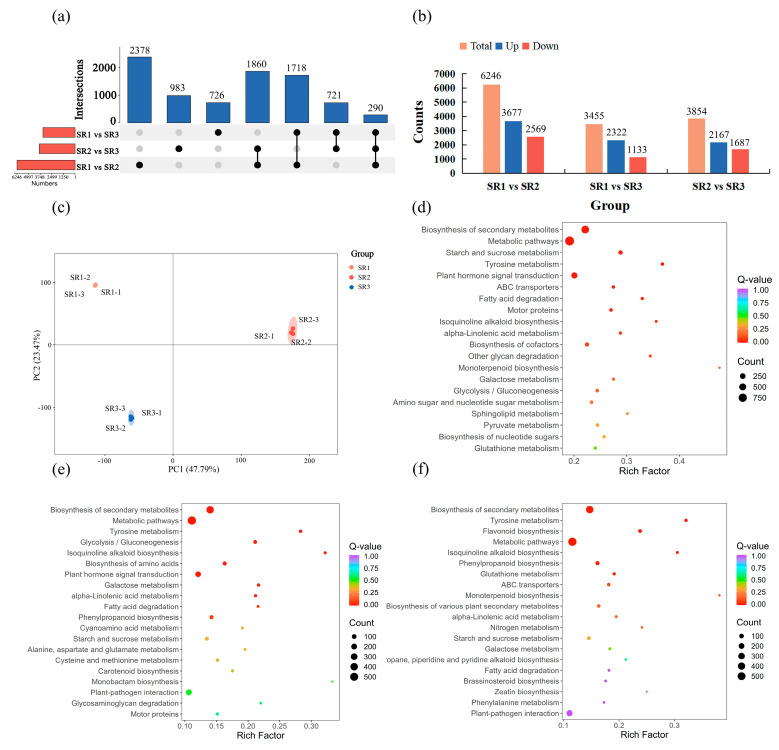
The identification and functional enrichment analysis of DEGs in three comparison groups. (**a**) UpSet plot of DEGs; (**b**) total, up-regulated, and down-regulated DEGs in SR1 vs. SR2, SR1 vs. SR3, and SR2 vs. SR3 groups; (**c**) principal component analysis of the DEGs; (**d**) KEGG enrichment of DEGs in SR1 vs. SR2 group; (**e**) The KEGG enrichment of DEGs in SR1 vs. SR3 group; (**f**) KEGG enrichment of DEGs in SR2 vs. SR3 group.

A total of 6246 DEGs (3677 up-regulated and 2569 down-regulated), 3455 DEGs (2322 up-regulated and 1133 down-regulated), and 3854 DEGs (2167 up-regulated and 1687 down-regulated) were found in SR1 vs. SR2, SR1 vs. SR3, and SR2 vs. SR3 group, respectively (Figure 3b). Among these, numbers of 2378, 726, and 983 DEGs were only identified from the SR1 vs. SR2, SR1 vs. SR3, and SR2 vs. SR3 groups, and the number of 290 DEGs was identified from three comparison groups (Figure 3a). Principal component analysis (PCA) results showed that the three treatment groups were clearly separated, with PC1 and PC2 accounting for 47.79% and 23.47% (Figure 3c). The DEGs were subjected to KEGG enrichment, and metabolic pathways and biosynthesis of secondary metabolites were mainly enriched among three groups. Furthermore, starch and sucrose metabolism, plant hormone signal transduction, and alpha-Linolenic acid metabolism in the SR1 vs. SR2 group; plant hormone signal transduction, alpha-Linolenic acid metabolism, and plant-pathogen interaction in the SR1 vs. SR3 group; and alpha-Linolenic acid metabolism and plant–pathogen interaction in the SR2 vs. SR3 group were the significantly enriched pathways (Figure 3d–f). These results suggested that the mentioned pathways might be involved in the adaptability of poplar largely at the transcriptional level.

### 2.4. The Analysis of Differentially Accumulated Metabolites

**Figure 4:**
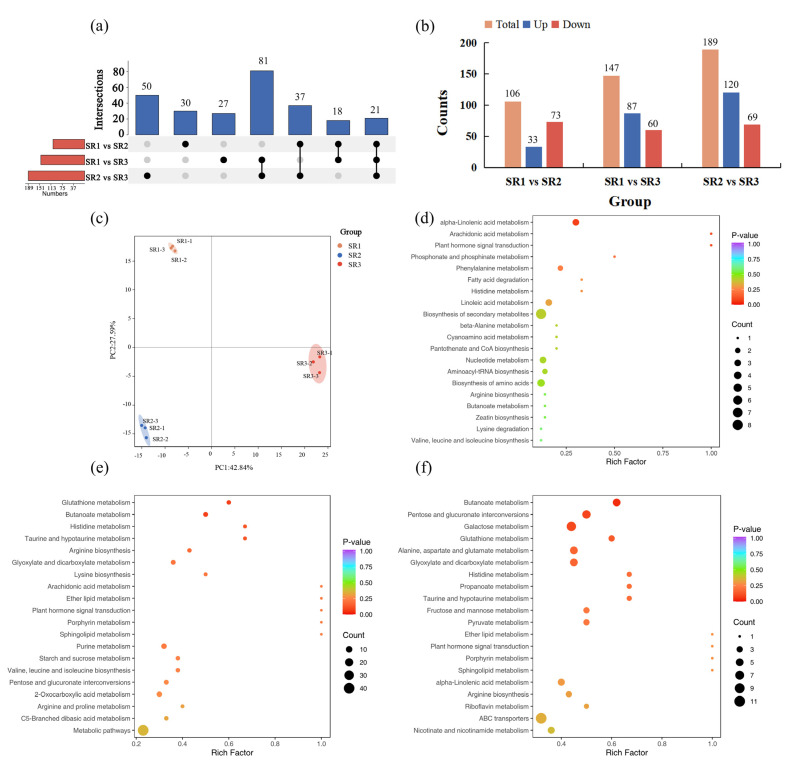
The identification and functional enrichment analysis of DAMs in three comparison group. (**a**) UpSet plot of DAMs; (**b**) total, up-accumulated, and down-accumulated DAMs in SR1 vs. SR2, SR1 vs. SR3, and SR2 vs. SR3 group; (**c**) principal component analysis of the DAMs; (**d**) KEGG enrichment of DAMs in SR1 vs. SR2 group; (**e**) KEGG enrichment of DAMs in SR1 vs. SR3 group; (**f**) KEGG enrichment of DAMs in SR2 vs. SR3 group.

A total of 106 DAMs (33 up-accumulated and 73 down-accumulated), 147 DAMs (87 up-accumulated and 60 down-accumulated), and 189 DAMs (120 up-accumulated and 69 down-accumulated) were found in the SR1 vs. SR2, SR1 vs. SR3, and SR2 vs. SR3 group, respectively (Figure 4b). Among these, numbers of 30, 27, and 50 DAMs were only identified from the SR1 vs. SR2, SR1 vs. SR3, and SR2 vs. SR3 groups, and the number of 21 DAMs was identified from the three comparison groups (Figure 4a). Principal component analysis (PCA) results showed that the three treatment groups were clearly separated, with PC1 and PC2 accounting for 42.84% and 27.59% (Figure 4c). The DAMs were also subjected to KEGG enrichment. Alpha-Linolenic acid metabolism in the SR1 vs. SR2 group; glutathione metabolism and butanoate metabolism in the SR1 vs. SR3 group; and alpha-Linolenic acid metabolism, glutathione metabolism, butanoate metabolism, and pentose and glucuronate interconversions in the SR2 vs. SR3 group were the significantly enriched pathways (Figure 4d–f). These results proposed that the mentioned pathways might be involved in the adaptability of poplar largely at the metabolic level.

### 2.5. The Analysis of Transcription Factors

**Figure 5:**
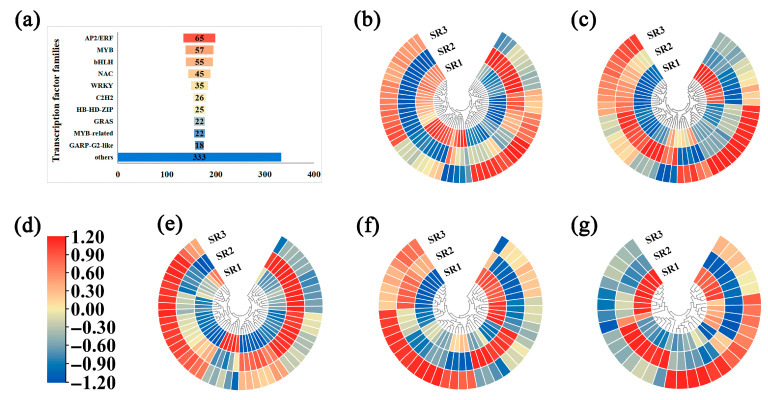
The analysis of transcription factors. (**a**) Number of transcription factors; (**b**) heat map of AP2/ERF family; (**c**) heat map of MYB family; (**d**) heatmap Legend: color scale from red to blue represents to the expression values from high to low, respectively; (**e**) heat map of bHLH family; (**f**) heat map of NAC family; (**g**) heat map of WRKY family.

The expression patterns of transcription factors in roots from three sites were analyzed. The results showed that AP2/ERF, MYB, bHLH, NAC, WRKY, C2H2, HB-HD-ZIP, GRAS, MYB-related, and GARP-G2-like families were identified with different expression patterns. The number of genes in AP2/ERF, MYB, bHLH, NAC, and WRKY families was over 30, that is, 65, 57, 55, 45, and 35, respectively (Figure 5a). Notably, SR3 showed significantly higher expression for most TF family genes than both SR1 and SR2. However, family-specific expression patterns were particularly evident. The majority of genes belonging to the WRKY family are highly expressed in SR1 but show lower expression levels in SR2 (Figure 5g, Table S3). Conversely, genes from the MYB and bHLH families are mostly expressed higher in SR2 and lower in SR1 (Figure 5c,e, Table S3). The number of up-regulated AP2/ERF and NAC family genes in SR1 and SR2 was basically similar (Figure 5b,f, Table S3). These differential expression profiles strongly suggest that these five TF families—particularly through their numerous differentially expressed members—play pivotal roles in mediating poplar’s transcriptional responses to varying environmental conditions.

### 2.6. The Expression Pattern of Genes Related to Antioxidant Enzymes and Stimulus Response

**Figure 6:**
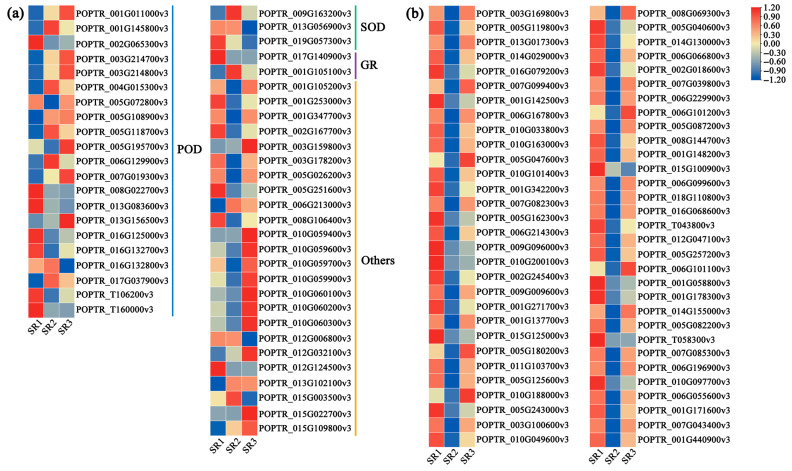
The expression patterns of DEGs related to antioxidant enzymes and stimulus response. (**a**) The heat map of the DEGs related to antioxidant enzymes. POD: peroxidase, SOD: superoxide dismutase, GR: glutathione reductase, Others: other genes related to antioxidant enzymes. (**b**) The expression patterns of DEGs related to stimulus response. The color scale from red to blue represents to the expression values from high to low, respectively.

The expression pattern of DEGs encoding antioxidant enzymes were explored. A total of 21, 3, and 1 DEGs related to POD (peroxidase), SOD (superoxide dismutase), and GR (glutathione reductase) were found, respectively (Figure 6a, Table S4). Most of the SOD genes were up-regulated in SR2. For POD genes, the number of up-regulated genes in three sites was almost equal. Other genes related to antioxidant enzymes are highly expressed in SR3. (Figure 6a, Table S4). Furthermore, DEGs involved in stimulus response were further identified. The results showed that almost all genes were up-regulated in SR1, which were down-regulated in SR2 (Figure 6b, Table S5). These results showed that genes related to POD, SOD, and stimulus response were associated with adaptability of poplar among three sites, especially in SR1 and SR2.

### 2.7. The Analysis of Genes Involved in Plant Hormone Signal Transduction

**Figure 7:**
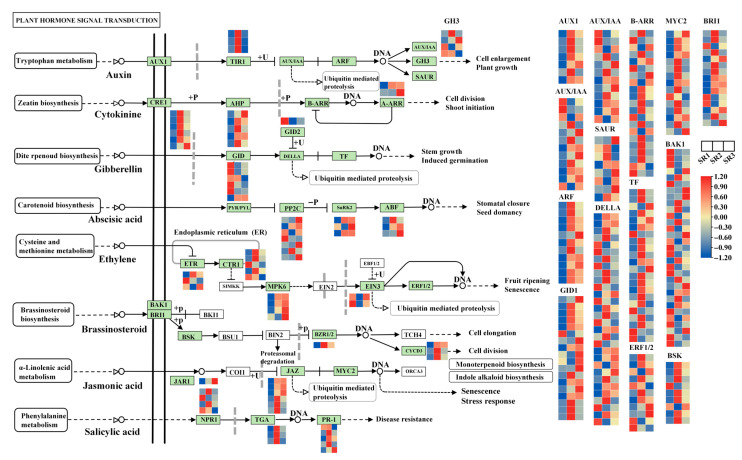
The expression of genes involved in plant hormone signal transduction. The color scale (red to blue) represents expression levels from high to low, respectively. The small rectangular elements represent genes (protein or mRNAs), with color coding denoting expression-level alterations. Green boxes: Significant differential expression (either up-regulation or down-regulation). White boxes: Non-significant expression changes. Circles denote metabolites (e.g., lipids/small molecules). In protein interaction networks: “+p (-p)”: Interactions involving phosphorylation (dephosphorylation) “+u”: Ubiquitination-related interactions. Solid arrow: indicates a direct and definite biochemical reaction or transformation. Dashed arrow: indirect link or unknown reaction. Blunt-end arrow (T-shaped): indicates inhibition, blockage, or negative regulation.

The expression patterns of DEGs involved in plant hormone signal transduction were analyzed. The results showed that the genes in the signal transduction pathways of auxin, cytokinine, gibberellin, abscisic acid, ethylene, brassinosteroid, jasmonic acid, and salicylic acid varied among three sites (Figure 7). In the auxin signal transduction pathway, *AUX1s*, *TIR1s*, *AUXs/IAAs*, and *ARFs* were highly expressed in SR2. In the cytokinine pathway, almost all *CRE1s*, *AHPs,* and *A-ARRs* were down-regulated in SR1. In the gibberellin pathway, a large number of *DELLAs* and *TFs* were highly expressed in SR2, and the large number of *BSK*, *BRT1*, *BZR1/2*, and CYCD3s were also highly expressed in SR2 in the brassinosteroid pathway. In the abscisic acid pathway, *PYRs*/*PYLs* were most up-regulated in SR1, while *SnRK2s* and *ABFs* were most down-regulated. In the jasmonic acid and salicylic acid pathway, a majority of *JAZs*, *MYC2s*, and *TGAs* were expressed lowly in SR1 (Figure 7, Table S6). These results demonstrated that the adaptability of poplar was partly determined by complicated plant hormone signaling network.

### 2.8. The Analysis of Genes and Metabolites Involved in Alpha-Linolenic Acid Metabolism

**Figure 8:**
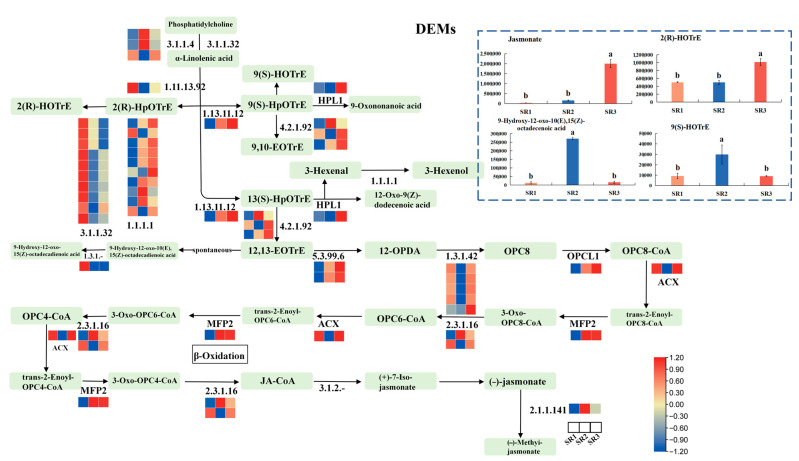
The genes and metabolites involved in the α-linolenic acid metabolism. The color scale from red to blue represents the expression values from high to low, respectively. The small green boxes represent compounds (other metabolites). To assess statistically significant differences, Duncan’s Multiple Range Test (IBM SPSS Statistics v26.0 software) was performed. Error bars represent the standard deviation of the means at *n* = 3. Significant differences (*p* < 0.05) are shown by distinct lowercase letters.

A total of 57 DEGs and four DAMs were identified from the α-linolenic acid metabolism pathway (Figure 8). The results showed that ten genes regulating Phosphatidylcholine to α-linolenic acid were up-regulated in SR1, while *OPCL1*, *MFP2*, genes regulating 12, 13-EOTrE to 12-OPDA, and genes regulating α-linolenic acid to 9(s)-HpOTrE or 13(S)-HpOTrE were down-regulated in SR1. All the five genes regulating 12-OPDA to OPC8, *HPL1,* and *ACXs* were down-regulated in SR2, while genes regulating (-)-jasmonate to (-)-Methy-jasmonate and *MFP2* were up-regulated in SR2 (Figure 8, Table S7). For differential metabolites, the relative content of jasmonate and 2(R)-HOTrE was highest in SR3, and the relative content of 9(s)-HOTrE, 9-Hydroxy -12-oxo-10(E), and 15(Z)-octadecenoic acid was highest in SR2 (Figure 8, Table S7). These results proposed that the α-linolenic acid metabolism pathway participated in regulating the environmental adaptability of poplar through the synthesis of jasmonate.

## 3. Discussion

As sessile organisms, trees have evolved sophisticated regulatory mechanisms to balance survival and growth under fluctuating environmental conditions [37]. Among their adaptive structures, fibrous roots are particularly responsive to subtle environmental variations, including changes in soil nutrient composition. This sensitivity makes them ideal systems for studying environmental adaptation. To elucidate these adaptive mechanisms, we employed an integrated transcriptomic and metabolomic approach that enables systematic identification of molecular responses to environmental gradients, facilitates network-level understanding of stress adaptation pathways, and provides location-specific analysis of root plasticity mechanisms. A total of 6246, 3455, and 3854 DEGs were identified from three comparison groups (Figure 3b). Among these DEGs, transcription factors including AP2/ERF, MYB, bHLH, NAC, and WRKY and others exhibited a significant proportion (Figure 5a), which are closely associated with environment responses. Specifically, AP2/ERF TFs coordinate plant stress tolerance through downstream gene regulation under various abiotic stresses including temperature extremes, drought, waterlogging, and salinity [38]. Similarly, bHLH transcription factors specifically regulate plant tolerance to abiotic stresses, which can also maintain the iron homeostasis under iron deficiency [39,40]. The extensively studied WRKY family plays multifaceted roles in stress adaptation, such as *PyWRKY71* in regulating *Populus yunnanensis* tolerance to admium stress, *PtWRKY39* in enhancing tolerance to drought and alkali salt [41,42], and additional functions in biotic stress defense against pathogens like *Alternaria* and insects including *Helicoverpa armigera* [43,44]. MYB family TFs demonstrate stress-specific regulatory patterns, with some members (e.g., PagMYB205) negatively influencing salt tolerance while others (PdMYB2R089/151) enhance drought resistance [45,46]. Furthermore, NAC transcription factors have been consistently implicated in regulating drought and salt tolerance mechanisms in poplar [47,48,49,50]. Our findings demonstrate that *(P. simonii × P. nigra) × P. deltoides* in SR3 exhibits a sophisticated transcriptional adaptation strategy, characterized by predominant upregulation of AP2/ERF, MYB, and bHLH family genes along with significant activation of WRKY and NAC members (Figure 5b,c,e,f; Table S3). This coordinated TF network activation strongly suggests SR3 represents a particularly challenging environment with limited nutrient availability and reduced precipitation, which collectively create substantial growth constraints. The observed transcriptional reprogramming reflects the poplar’s adaptive response to these stressful conditions, where multiple stress-responsive pathways are simultaneously engaged to maintain physiological homeostasis. These results not only provide molecular insights into poplar’s environmental adaptation mechanisms but also establish a robust foundation for future studies aimed at elucidating the precise regulatory networks underlying stress resilience in perennial woody species.

The study revealed that *(P. simonii × P. nigra) × P. deltoides* in SR2 exhibits a unique antioxidant defense strategy characterized by coordinated expression patterns of key enzymatic components; these plant enzymes include POD, SOD, GR, and others [51,52]. Specifically, the expression of SOD genes was predominantly upregulated. These genes catalyze the critical first step in reactive oxygen species (ROS) scavenging and serve as biomarkers for oxidative stress resistance, while GR genes were concomitantly downregulated (Figure 6a, Table S4). GR is known to be regulated by various environmental stresses and contributes to stress resistance in some plants [53,54,55]. Furthermore, DEGs related to stimulus response were down-regulated in SR2 (Figure 6b, Table S5). These results suggest that poplars in SR2 may have evolved an efficient SOD-dominated antioxidant system that preemptively neutralizes oxidative stress, thereby reducing the need for both GR activity and stimulus perception pathways. This adaptive mechanism provides insights into how perennial plants optimize their defense systems under specific environmental constraints.

Plant hormones coordinate complex metabolic reprogramming to enhance stress acclimation, as demonstrated by extensive genetic studies of hormone biosynthesis and signaling mutants [56,57]. Three key hormonal interactions emerge as particularly critical: the gibberellin has a regulatory effect on plant development, especially for root; the auxin can regulate the expression of gibberellin synthesis-related genes, while gibberellin is involved in the transport of polar auxin [58,59,60]; and brassinosteroids are also closely related to auxin through modulating the auxin transport [61]. In this study, *AUX1s*, *TIR1s*, *AUXs/IAAs*, and *ARFs* in the auxin signal pathway; most of the *DELLAs* and *TFs* in the gibberellin pathway; and most of *BSK*, *BRT1*, *BZR1/2*, and *CYCD3s* in the brassinosteroid pathway were highly expressed in SR2 (Figure 7, Table S6), indicating a positive regulatory effect on plant growth. These results could explain the high tree height and diameter of breast height of (*P. simonii* × *P. nigra*) × *P. deltoides* in SR2 powerful. The integration of jasmonate and salicylic acid signaling pathways forms a critical defense network against biotic stressors, where jasmonate mediates insect and pathogen defense responses [57], while salicylic acid induces systemic acquired resistance to diverse pathogens. In this study, a great number of *JAZs* and *MYC2s* in the jasmonic acid pathway and *NPR1s* and *TGAs* in the salicylic acid pathway were expressed lowly in SR1 while being highly expressed in SR2 (Figure 7, Table S6). This differential expression pattern demonstrates that SR2 poplars develop a more efficient environmental adaptation strategy through coordinated activation of dual defense pathways. The findings provide molecular-level insights into the remarkable biotic stress adaptation advantages observed in *(P. simonii × P. nigra) × P. deltoides* from the SR2 region.

Metabolomics involves comprehensive analyses of metabolites in plant tissues and cells. These metabolites include sugars, amino acids, organic acids, secondary metabolites (e.g., alkaloids and flavonoids), lipids, and more [62]. Lipids encompass a wide range of compounds, such as fatty acids, glycerol-phospholipids, galactolipids, sphingolipids, and neutral lipids. As primary biomolecules, lipids play major roles in structural components, sustainable carbon and energy storage, active signal transducers, and surface coverings [63]. Additionally, lipids are one of the most important components of biofilms in all plant tissues, and membrane lipid remodeling is one of the effective adaptation strategies for plants to resist various abiotic stresses [64,65,66]. α-linolenic acid is a component of plant cell membrane, which is involved in the biosynthesis of jasmonic acid. The content of jasmonate in SR3 was higher than SR1 and SR2 (Figure 8, Table S7), further indicating that SR3 was a severe condition with lots of stress for (*P. simonii* × *P. nigra*) × *P. deltoides.*

**Figure 9:**
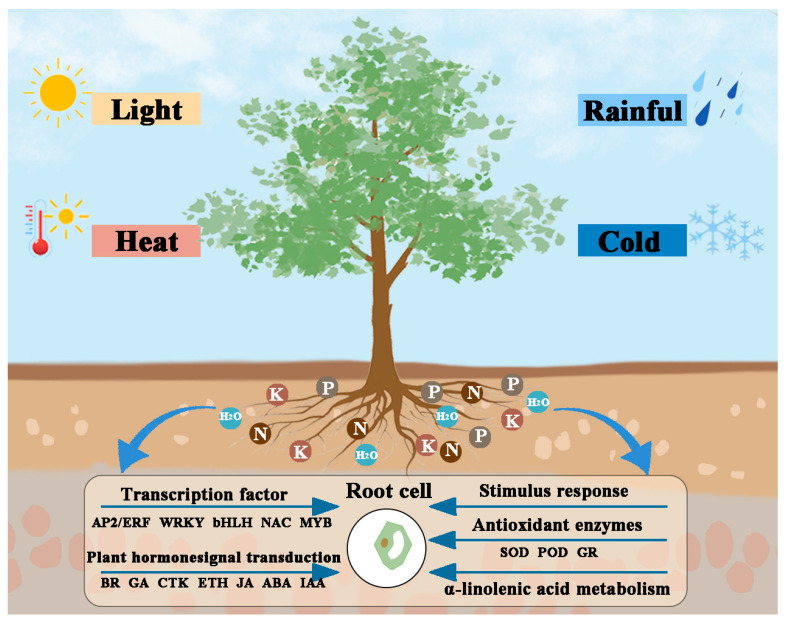
The model of environmental adaptability.

In summary, the wind speed differences among the three sites were not significant, indicating that wind speed was not a primary factor influencing growth variation. SR1 exhibited the highest relative humidity at 61.88%, while SR2 had higher temperature, precipitation, and soil nutrient levels compared to the other two sites. Correspondingly, *(P. simonii × P. nigra) × P. deltoides* planted in SR2 demonstrated superior growth performance, with both tree height and diameter at breast height (DBH) outperforming those at the other sites. In-depth transcriptomic and metabolomic analyses revealed that the exceptional performance of SR2 plants was closely associated with differential expression in their antioxidant enzyme systems, plant hormone signal transduction, α-linolenic acid metabolism, and key transcription factors (AP2/ERF, MYB, bHLH, WRKY, and NAC) (Figure 9). Although current research highlights the growth advantages of SR2, its long-term suitability may require further validation under interannual climate fluctuations (e.g., drought, cold stress). Similarly, the outstanding growth performance of *(P. simonii × P. nigra) × P. deltoides* in the SR2 site could also be influenced by unmeasured variables, such as photosynthetic rate (Pn), stomatal conductance (gs), transpiration rate (ET), soil water content (SWC), and rhizosphere microbial community structure [67].

Plant genotype–environment (G×E) interactions are orchestrated through hierarchical regulatory cascades initiated by environmental stressors. These cascades activate core transcription factors, inducing stress-responsive genes (e.g., OsNAC6) [68] while establishing transgenerational stress memory via epigenetic marks (DNA methylation, H3K27me3) [69]. Concurrent metabolic reprogramming sustains osmotic homeostasis via osmoprotectant accumulation and oxylipin signaling (e.g., jasmonates), regulated by ABA/JA/SA crosstalk through the PYR-PP2C-SnRK2 module [70]. From a genetic perspective, genome-wide association studies (GWAS) and genotype-by-environment interaction analyses (GWEIS) identify environment-dependent loci, providing candidate targets for mechanistic validation [71]. Current research on genotype-by-environment (G × E) interactions faces key challenges, including unclear organ-specific responses, complex stress interactions, and difficulties translating lab findings to real-world applications [72]. Future research advancements will depend on three key approaches: multi-omics technologies to elucidate regulatory mechanisms in plant-microbe symbiosis [73], CRISPR/Cas9 genome editing for functional characterization of adaptive genes [74], and environmental simulation platforms integrated with transcriptomic profiling to unravel the molecular basis of phenotypic plasticity [75]. Collectively, these methodologies provide crucial scientific foundations for developing climate-resilient crop varieties, establishing precision agriculture systems, advancing adaptive forest management practices, and formulating effective climate change mitigation strategies.

## 4. Materials and Methods

### 4.1. Plant Materials

The (*P. simonii* × *P. nigra*) × *P. deltoides* clones were planted at state-owned Yushutai Forest Farm in Lishu, Jilin Province; Xinmin Machinery Forest Farm in Xinmin, Liaoning Province; and Cuohai Forest Farm in Longjiang, Heilongjiang Province, China. In the spring of 2017, one-year-old bare-root seedlings were established at 2 × 4 m. Three experimental forests were designed using a randomized complete block design, with six plants per block and five replications. A single row of protection trees, *P. simonii* × *P. nigra*, was set between each block, and two rows were set around the forests. A uniform experimental management protocol was implemented across all three study sites. During the first year post-planting, standardized manual irrigation and physical weed control methods were applied. All anthropogenic interventions were terminated after the first year, with no fertilization applied at any site. The information of the three sites and the growth traits of six-year-old poplar were listed in Table 1. In July 2022, the same length fibrous roots of six-year-old plants from Lishu (named SR1), Xinmin (named SR2), and Cuohai (named SR3) were collected with liquid nitrogen for the transcriptome and metabolome analysis.

### 4.2. The Determination of Soil Element Content

In July 2022, composite soil samples from all three study sites (SR1, SR2, and SR3) were collected, air-dried, and homogenized for analysis of total nitrogen (TN), potassium (TK), and phosphorus (TP) content (five independent sampling points were systematically established at each study site, with three biological replicates measured per sampling point). Nutrient quantification was performed by Shanghai Enzyme-linked Biotechnology Co., Ltd. (Shanghai, China) using standardized methods with three technical replicates. The total nitrogen (TN) content was detected using the Kjeldahl method [76], total potassium (TK) content was detected using flame spectrophotometry [77], and total phosphorus (TP) was determined using the trace method [78,79].

### 4.3. Total RNA Isolation, Library Construction, and Transcriptome Sequencing

Total RNA was extracted using the modified CTAB method with three biological replicates [80]. RNA concentration was analyzed by the Qubit^®^ RNA Assay Kit on a Qubit^®^ 2.0 Fluorometer (Thermo Fisher Scientific, Waltham, MA, USA), and RNA integrity was evaluated by the RNA Nano 6000 Assay Kit of the Bioanalyzer 2100 system (Agilent Technologies, Santa Clara, CA, USA). RNA samples with RIN ≥ 7.0 were used to construct strand-specific cDNA libraries, which were sequenced on the Illumina NovaSeq 6000 platform (150 bp paired-end) by Metware Biotechnology Co., Ltd. (Wuhan, China).

### 4.4. Transcriptome Analysis

Fastp was used to filter out adapters and low-quality data [81], which were aligned to a *Populus simonii* reference genome (sequenced and assembled in-house, unpublished) using HISAT2 [82]. StringTie was used for novel gene prediction, and the transcription factors were predicted using iTAK [83,84,85]. FeatureCounts were used to calculate the gene read counts, and the FPKM (Fragments Per Kilobase of transcript per Million fragments mapped) was obtained based on the gene length [86]. DESeq2 was used to analyze DEGs with |log2Fold Change| ≥ 1 and a false discovery rate < 0.05. The genes were annotated using Nr (National Center for Biotechnology Information non-redundant), KEGG (Kyoto Encyclopedia of Genes and Genomes), and Gene Ontology (GO) databases. For heatmap visualization, log2-transformed average FPKM values from three biological replicates were normalized using row scaling in TBtools (v2.018) [87].

### 4.5. Sampling Preparation and Metabolite Extraction

The samples were vacuum-freeze-dried using a Scientz-100F lyophilizer (Ningbo Scientz Biotechnology Co., Ltd., Ningbo, China) and then ground to powder (30 Hz, 1.5 min) with a Retsch MM 400 grinder. Exactly 50 mg of powder was extracted with 1200 μL of ice-cold 70% methanol aqueous solution containing internal standards. The mixture was vortexed for 30 s at 30 min intervals (6 cycles total), with storage on ice between vortexing steps. After centrifugation (12,000 rpm, 3 min, 4 °C), the supernatant was filtered through a 0.22 μm membrane for UPLC-MS/MS analysis (Ultra Performance Liquid Chromatography–Tandem Mass Spectrometry) by Metware Biotechnology Co., Ltd. (Wuhan, China). The UPLC conditions were identical to those described by Li et al. [88,89].

### 4.6. Metabolome Analysis

The MS data were analyzed using Analyst 1.6.3 software [84,90]. Qualitative and quantitative characterization of metabolites was performed based on MWDB (Metware Database). The DAMs were identified with VIP > 1 and (fold change ≥ 2 or ≤ 0.5) (*p* < 0.05, FDR-adjusted *p*-values). Subsequent pathway enrichment analysis was conducted by mapping DAMs to KEGG pathways (version 2023) using the KEGG database.

### 4.7. qRT-PCR Verification

Six DEGs were randomly selected for RNA-seq validation by qRT-PCR using *PsnActin* as the internal control. Total RNA, extracted and provided by Metware Biotechnology Co., Ltd. (Wuhan, China), was reverse-transcribed into cDNA using the PrimeScript™ RT Reagent Kit (TaKaRa, Dalian, China). qRT-PCR was performed on the CFX Opus 96 system (Bio-Rad Laboratories, Hercules, CA, USA) with TB Green^®^ Premix Ex Taq^TM^ II (TaKaRa, Dalian, China) under the following conditions: 95 °C for 30 s, followed by 40 cycles of 95 °C for 5 s and 60 °C for 30 s. The relative expression level for DEGs was calculated using the 2^−ΔΔCT^ method [91]. Primer sequences were listed in Table 2.

## 5. Conclusions

From the three studied sites, SR2 was found to be the appropriate cultivation area for the (*P. simonii × P. nigra*) × *P. deltoides* clone, with higher soil nutrient content, average temperature (8.2 °C vs. 6.9 °C/3.4 °C), and greater precipitation (600 mm vs. 480 mm/465 mm). Transcriptomic and metabolomic profiling revealed substantial molecular differentiation; a total of 6246, 3455, and 3854 DEGs and 106, 147, and 189 DAMs were found in the SR1 vs. SR2, SR1 vs. SR3, and SR2 vs. SR3 groups. DEGs, determining the adaptability, were primarily associated with the antioxidant enzyme system, stimulus response, α-linolenic acid metabolism (57 DEGs), and plant hormone signal transduction pathways, while DAMs were primarily associated with α-linolenic acid metabolism (4 DAMs), glutathione metabolism, butanoate metabolism, and pentose and glucuronate interconversions. Notably, α-linolenic acid metabolism emerged as a central regulatory hub, potentially coordinating jasmonate-mediated stress responses, membrane lipid remodeling, and energy homeostasis. Furthermore, five transcription factor families (AP2/ERF, MYB, bHLH, NAC, and WRKY) exhibited pronounced differential expression patterns, suggesting their pivotal roles in environmental signal perception, stress-responsive gene regulation, and growth-defense balance modulation. These results collectively demonstrate that the climatic conditions in SR2 promote optimal molecular adaptation in poplar through coordinated regulation of stress-response pathways and developmental processes. Future research will build upon this foundation by integrating environmental simulation with gene expression analysis, leveraging multi-omics technologies (such as transcriptomics, proteomics, and metabolomics) and CRISPR/Cas9 gene editing to elucidate the mechanisms of gene–environment interactions, thereby enabling more precise molecular breeding and adaptive improvement studies.

## Figures and Tables

**Table 1:** The information of three sites and the growth traits of six-year-old poplar.

Site	Longitude	Latitude	Average Temperature	Precipitation	Wind Speed	Relative Humidity	Tree Height	Diameter of Breast Height
SR1 (Lishu)	124°54′	43°62′	6.9 °C	480 mm	3.83 m/s	61.88%	16.00 m	15.90 cm
SR2 (Xinmin)	122°56′	41°88′	8.2 °C	600 mm	3.56 m/s	57.08%	17.73 m	19.33 cm
SR3 (Cuohai)	122°85′	47 °45′	3.4 °C	465 mm	3.52 m/s	56.54%	13.15 m	13.32 cm

**Table 2:** The primer sequences used for qRT-PCR verification.

Gene ID	Forward Primer (5′-3′)	Reverse Primer (5′-3′)
POPTR_001G271700v3	CAGCCAATGAGGCCTGTTGGTG	GACTCATCAACTGATTGCTCGGCA
POPTR_001G281900v3	TCCGTCTAGCAGTGAGAGATCTTCA	GCAATTGAACCGGTCTCTCTGC
POPTR_003G011200v3	TTCCACCGTCGTCGACTCTG	CACGAACAGAAGTCATGCCGA
POPTR_009G096000v3	CCAGTACTCCTTCGGGATCTGA	CCAACTGTCCAGACTCCATCC
POPTR_014G020500v3	TGGAAATTGTAATGGAAGAGGAGGGA	GCCCTGACCTAACATCTACTTCTCTA
POPTR_019G037400v3	ATGGCAAATTACGCACCGGGAA	CGAAGCAGTTTGATCTCACGAAGG
*PsnActin*	CCAGCAACCGCAATACAA	CTTCACCATTCCAGTTCCATT

## Data Availability

The raw data supporting the conclusions of this article will be made available by the authors on request.

## Supplementary Materials

The following supporting information can be downloaded at: https://www.mdpi.com/article/10.3390/plants14172691/s1, Table S1: The quality of transcriptome data; Table S2: Functional annotations of new genes; Table S3: The FPKM of exhibited transcription factors; Table S4: The FPKM of DEGs related to antioxidant enzymes; Table S5: The FPKM of DEGs related to stimulus response; Table S6: The FPKM of DEGs involved in plant hormone signal transduction; Table S7: The FPKM of DEGs and the relative content of DAMs in α-linolenic acid metabolism.
